# Carcinogenesis from chronic exposure to radio-frequency radiation

**DOI:** 10.3389/fpubh.2022.1042478

**Published:** 2022-10-31

**Authors:** James C. Lin

**Affiliations:** Departments of Electrical and Computer Engineering, Bioengineering, and Physiology and Biophysics, University of Illinois Chicago, Chicago, IL, United States

**Keywords:** wireless and mobile technology, exposure safety and regulations, cancer risk, human-agent interaction, animal experiment, ALARA (as low as reasonably achievable)

## Abstract

The past two decades have seen exponential growth in demand for wireless access that has been projected to continue for years to come. Meeting the demand would necessarily bring about greater human exposure to microwave and radiofrequency (RF) radiation. Our knowledge regarding its health effects has increased. Nevertheless, they have become a focal point of current interest and concern. The cellphone and allied wireless communication technologies have demonstrated their direct benefit to people in modern society. However, as for their impact on the radiation health and safety of humans who are unnecessarily subjected to various levels of RF exposure over prolonged durations or even over their lifetime, the jury is still out. Furthermore, there are consistent indications from epidemiological studies and animal investigations that RF exposure is probably carcinogenic to humans. The principle of ALARA—as low as reasonably achievable—ought to be adopted as a strategy for RF health and safety protection.

## Introduction

Microwave and radiofrequency (RF) radiations power all over-the-air wireless channels, communication links, and network systems through which text, files, images, and videos are transferred by mobile devices and related platforms. The recent decades have seen exponential expansion in popularity for mobile access that has been forecasted to persist in the foreseeable future. Satisfying the demand would necessarily bring about greater human exposure to microwave and RF radiation.

Asides from primary intended roles as a carrier or infrastructure that enables the communication technology, microwave and RF radiation may induce additional effects that could influence the vital functions of living organisms. The biological changes caused would manifest in multiple physical and biological spheres. They may or may not be grossly apparent or observable soon after exposure of the living organisms. In some cases, they may only manifest until years later—they may develop years to decades after repeated low-level exposures.

The health impact of RF and microwave radiations has been a subject of scientific investigation shortly after demonstration of their production in scientific laboratories, over a century ago (1, 2). Without any doubt, their use especially in cellular mobile communication and associated wireless technologies have enriched human lives. Our knowledge regarding its health effects has increased gradually. Nonetheless, RF radiation has come to be a focal point of interest as a result of accelerated use of RF and microwave radiation in wireless mobile communications.

The WHO's International Agency for Research on Cancer (IARC) categorized exposure to RF and microwaves as a possible carcinogen to humans in 2011. The IARC had evaluated existing scientific studies and came to the conclusion that while data was imperfect and restricted, particularly regarding reports from animal experiments, epidemiological investigations concerning elevated risks for gliomas (a type of malignant brain cancer) and acoustic neuromas (a non-malignant tumor of the acoustic or vestibulocochlear nerves) among heavy users or long-term customers of cellphones, are satisfactorily robust to warrant a classification of possibly carcinogenic in humans for exposure to RF and microwave radiation (3, 4).

Recently, two commonly distributed RF health protection recommendations revised their guidelines and standards. The updated International Commission on Nonionizing Radiation Protection guidelines (5) and Institute of Electrical and Electronic Engineers International Committee on Electromagnetic Safety standards (6) are strongly connected to acute temperature rises induced by RF heating inside the human body. The updated safety guidelines and standards showed without any question the groups' staunch convictions of nothing but heat to worry about with microwave and RF radiation.

A persistent and vexing question lingers concerning these guidelines and standards for safe long-term exposure to RF radiation (in contrast to exposures shorter than 6 or 30 min). A general sense on the absence of appreciation of scientific evidence regarding long-term exposure below the basic restrictions continues with these safety guidelines and standards.

There is also the question of how there can be such divergent evaluations and inferences of the identical scientific findings by WHO's IARC, ICES, and ICNIRP. To be fair, scientists are not impervious to conflicts of interest such as conflicting financial interests or personal relations which could affect the deliberations and reporting through such experiences as groupthink. Also, in some ways, it may parallel the compulsion by big business to choose profit over societal concerns—big businesses often use a range of organized and refined tactics to enhance and protect their commercial interests, and regrettably in some cases these tactics come at the expense of public health.

Human beings repeatedly render decisions and select choices that challenge principled logic. Indeed, science has not been devoid of politics—weird as that may sound. Various biases can impair sensible reasoning and result in bad judgments. Groupthink can mislead human beings and inhibit scientists from making understandable inferences. Regrettably, groupthink or herd mentality is as rampant today as ever. Has science become partisan? And if science becomes partisan, is it science or politics, or would it be political science? At times, science gets wrapped up in politics and politics intervenes with science. It may simply turn out to be a matter of guilelessly being politically correct of the willing. Less than rigorous enforcement of policies in research conduct or full disclosure of financial conflicts can lead to failures in guiding and informing the development of transparent and trustworthy evaluations of scientific evidence for safety protection. Scientists may not always be consistent, coherent, or as transparent as promoted.

## Carcinogenicity in rats exposed lifelong to RF radiation

Briefly, through 2016, counting all carcinogenicity and co-carcinogenicity investigations in laboratory rats experimentally exposed life-long or for a minimum of 2 years to cellphone-type RF radiation, there were 9 reported studies revealing important modification in incidence of cancer rates between RF- and sham-exposures (7). The RF exposures involved frequencies ranging from 836 to 2,450 MHz and used common wireless mobile-phone modulations and telecommunication protocols. In summary, there were more reported results showing no cancer-causing responses than showing cancer effects, regardless of the study design, merit, flaws, experimental quality, shortcomings, limitations, or methodological weaknesses. Note that many investigations were performed by restraining the animals during exposure, which included the observation of an apparent protective (tumor-inhibiting) response. Restraining a rodent causes stress for the animal and the stress response interferes with neoplastic development (8, 9). Furthermore, most of them are one-of-a-kind investigations—few studies were conducted as an extant, independent replication or confirmation. The study reports have been inconsistent and fraught with omissions of experimental details in some cases. Thus, it is challenging to make an unequivocal conclusion. A remarkable flaw was that many of the reported projects did not involve concurrent or cage-control animals as part of the experimental protocol, or relevant data were not included in the statistical analyses.

Whether RF exposure from wireless and mobile devices and systems poses a personal health risk has been a vexing question for some time. The answer has been equivocal and controversial. The effect of RF exposure on carcinogenicity thus remained tentative, as noted in the IARC report. The inconsistencies and ambiguities present uncertainties to estimation of risks of exposure to RF radiation from cellphones to public health.

## Recent results from laboratory rat experiments

It is noteworthy that the results from animal experiments that were coveted by IARC at the time of its classification were supplied by the National Toxicology Program (NTP) of the U.S. National Institute of Environmental Health Science (NIEHS) in 2018. Specifically, NTP/NIEHS presented the findings of two types of cancers in laboratory rats exposed, lifelong, to RF radiation that were commonly used for 2G and 3G cellphone operations (10, 11). The study was the largest animal health effect study taken upon by NTP/NIEHS in its history, including the large number of toxic chemical agents. The results showed, among other findings, there was statistically significant and clear evidence (compared to concurrent controls) that exposure to cellphone RF radiation had caused the observation of malignant cardiac schwannoma (a rare form of tumor) in male rats whose RF-induced body temperature elevation was up to 1°C. Furthermore, the same schwannoma risk was indicated among female rats. NTP also reported unusual patterns of damage to heart tissue (cardiomyopathy) in both RF-exposed males and females when compared with concurrent control rats. Based on statistical significance, the outcome of pathological examinations showed some signs of RF-dependent cancer-causing activity in the male-rat brains, malignant gliomas in particular. Outcomes for females were regarded as ambiguous for gliomas compared to concurrent controls.

Moreover, results from extensive research of carcinogenesis in rats exposed to 3G, 1800-MHz RF radiation performed by Ramazzini Institute's Cesare Maltoni Cancer Research Center in Bologna, Italy was published soon after the NTP/NIEHS report (12). The investigation involved whole-body exposure of male and female rats, either prenatal until death or lifelong, under far zone plane-wave exposure conditions. During the 19-h per day exposures for ~2 years, the whole-body SARs were 0.001, 0.03, and 0.1 W/kg. A statistically significant rise was observed for cardiac schwannomas incidents in males for whole-body RF exposure at 0.1 W/kg. It is noteworthy that the NTP/NIEHS and Ramazzini RF exposure research showed comparable findings of cardiac schwannomas and cerebral gliomas. Thus, two comparatively well-conducted animal investigations using the same strain of rats demonstrated consistent outcomes in significantly elevated cancer risks.

## Safety protection guidelines and standards

While recognizing that the two recent large animal studies employed good-laboratory practices (GLP), and prolonged exposures of rats for their entire lifespan, the current revisions of safety protection guidelines and standards decided to nitpick with objections based on “chance differences” and exposed rat core-body temperatures of up to 1°C at 0.1 W/kg. Oddly, in choosing to do so, ICES (6) and ICNIRP (5) neglected the incongruity of suggesting a 1°C body-core temperature elevation as the putative cancer-causing agent. Furthermore, the recommendations overlooked entirely the consequences of RF exposures (the independent variable for the animal experiments) or preferred evading the implications through pretenses which may be paraphrased as “the evidence or findings do not provide credible indication of adverse effects caused by chronic RF exposures” ICES (6) and ICNIRP (5). These same groups proceeded to use ambiguous expressions such as “substantial limitations” to assert the motives in barring any “conclusions being drawn concerning RF EMFs and carcinogenesis,” to defend the revised RF safety protection guidelines and standards. Evidently, the revisions were predicated on the groups' strong convictions of nothing but heat to worry about with microwave and RF radiation.

Moreover, it opined that although epidemiological studies of RF radiation associated with cellphone use and cancer risk have been performed, reported results from research on increased acoustic neuroma, brain gliomas, meningioma, and parotid gland tumors have not provided sufficient evidence of greater cancer risk. The recently revised safety guidelines and standards also noted that while somewhat elevated odds ratios were observed, there are inconsistencies and limitations such as recall or selection bias which preclude the epidemiological results from being considered for recommending exposure guidelines and standards. The predilection to reject and disparage positive outcomes, and affection for and eagerness to accept negative conclusions, all at once, are palpable and concerning.

## Discussion and conclusion

The recently revised ICES standards and ICNIRP safety guidelines make recommendations to supposedly guard against known hazardous health consequences in humans resulting from exposure to RF frequencies up to 300 GHz. The guidelines and standards are for short-term exposures of 6–30 min, based on limiting whole-body temperature from increasing above 1°C or local tissue temperature to 5°C (5, 6). The updated safety guidelines and standards demonstrate without any doubt the groups' strong convictions on nothing but heat to worry about with RF radiation.

If the groups that promulgate the safety protection recommendations assume what seems to be their stance regarding experimental results in rats by U.S. NTP/NIEHS that a whole-body temperature increase of 1°C causes cancer, then the safety or reduction factors of 50 recommended for the general population, or 10 for occupationally engaged working person would be borderline for the specified objective and practically worthless from the standpoint of protecting “safety.” It is noteworthy that the highest SAR or exposure level chosen by NTP/NIEHS that showed increased carcinogenicity in rats is essentially the same as that chosen by ICES and ICNIRP for their basic restrictions.

The fact is that the missing pieces according to IARC (4), or the previously coveted experimental animal data (7) as currently provided by NTP/NIEHS (10) and Ramazzini Institute (12) complement IARC's evaluation of human epidemiological studies in support of its classification of RF radiation as a possible carcinogen. It gives rise to the plausibility for IARC to enhance its previous, mostly epidemiology-based classification to the higher level of “probably cancer causing” for RF exposure.

Furthermore, more recent systematic reviews and meta-analyses of the case-control research on mobile phone use have reported statistically significant increases in brain tumor risk associated with 1,000 or more hours of cellphone use, or about 17 min per day over 10 years (13, 14).

Off-the-shelf cellphones have SAR ranging from 0.2 and 0.5 W/kg (15). The U.S. Federal Communications Commission (FCC) rules require that they do not exceed the SAR limit of 1.6 W/kg for cellphones operating at its highest possible power level. Clearly, cellphones are operating at a fraction of the SAR acceptable to FCC and they are well below (e.g., only 10%) the 2.0 W/kg promulgated by ICES and ICNIRP. It is meaningful to mention that presently allowable power for cellphones are roughly 5 orders of magnitude higher than a prototype cellphone consuming 3.5 μW of power to enable voice calls by harvested ambient RF power (16). It is conceivable that upcoming developments would enable cellphone functions including data transmission via energy harvesting. Obviously, it stays important to be attentive so that the supporting ambient RF radiation will not create a cause of safety and health concern.

The simple and effective public health notion of “An ounce of prevention is better than a pound of cure” may conjure up old fashioned. It may arouse intense reactions, with enormous defiance especially from individuals who may be beneficiary of modern promotions. The cellphone and allied wireless communication technologies have shown their direct benefit to people in modern society. However, as for their impact on the radiation health and safety of humans who are subjected unnecessarily to various levels of RF exposure over prolonged durations or even over their lifetime, the jury is still out. Furthermore, there are consistent indications from epidemiological studies and animal investigations that RF exposure is, at least, probably carcinogenic to humans. The principle of ALARA—as low as reasonably achievable—ought to be adopted as a strategy for RF health and safety protection.

## Data availability statement

The original contributions presented in the study are included in the article/supplementary material, further inquiries can be directed to the corresponding authors.

## Author contributions

The author confirms being the sole contributor of this work and has approved it for publication.

## Funding

This work was supported by DSC-08034011324PRD.

## Conflict of interest

The author declares that the research was conducted in the absence of any commercial or financial relationships that could be construed as a potential conflict of interest.

## Publisher's note

All claims expressed in this article are solely those of the authors and do not necessarily represent those of their affiliated organizations, or those of the publisher, the editors and the reviewers. Any product that may be evaluated in this article, or claim that may be made by its manufacturer, is not guaranteed or endorsed by the publisher.

## References

[1] Schwan HP. Electrical properties of tissue and cell suspensions. Adv Biol Med Phys. (1957) 5:147–209. 10.1016/B978-1-4832-3111-2.50008-0 13520431

[2] Schwan HP. Early history of bioelectromagnetics. Bioelectromagnetics. (1992) 13:453–67. 10.1002/bem.2250130604 1482412

[3] Baan R Grosse Y Lauby-Secretan B El Ghissassi F Bouvard V Benbrahim-Tallaa L. Carcinogenicity of radiofrequency electromagnetic fields. Lancet Oncol. (2011) 12:624–6. 10.1016/S1470-2045(11)70147-4 21845765

[4] IARC. Working group on the evaluation of carcinogenic risks to humans non-ionizing radiation, Part 2: radiofrequency electromagnetic fields. IARC Monogr Eval. Carcinog Risks Hum. (2013) 102:1–460. 24772662 PMC4780878

[5] ICNIRP. Guidelines for limiting exposure to electromagnetic fields (100 kHz to 300 GHz). Health Phys. (2020) 118:483–524. 10.1097/HP.0000000000001210 32167495

[6] ICES. IEEE Standard for Safety Levels With Respect to Human Exposure to Electric, Magnetic, and Electromagnetic Fields, 0 Hz to 300 GHz," (Revision of IEEE Std C95.1-2005/ Incorporates IEEE Std C95.1-2019/Cor 1-2019). (2019). p. 1–312.

[7] Lin JC. Cancer occurrences in laboratory rats from exposure to rf and microwave radiation. IEEE J Electromagnet. (2017) 1, 2–13. 10.1109/JERM.2017.2721427

[8] Frick LR Arcos ML Rapanelli M Zappia MP Brocco M Mongini C. Chronic restraint stress impairs T-cell immunity and promotes tumor progression in mice. Stress. (2012) 12:134–43. 10.1080/10253890802137437 18609297

[9] Feng Z Lianxin Liu L Zhang C Zheng T Wang J Lin M. Chronic restraint stress attenuates p53 function and promotes tumorigenesis. PNAS. (2009) 109:7013–8. 10.1073/pnas.1203930109 22509031 PMC3345015

[10] NTP/NIEHS. Technical Report on the Toxicology and Carcinogenesis Studies in HSD: Sprague–Dawley SD rats Exposed to Whole-Body Radio Frequency Radiation at a Frequency (900 MHz) and Modulations (GSM and CDMA) Used by Cell Phones, NTP Tech. Rep. 595, Raleigh, NC (2019). 10.22427/NTP-TR-595 PMC8039879 33562898

[11] Wyde ME Horn TL Capstick MH Ladbury JM Koepke G Wilson PF. Effect of cell phone radiofrequency radiation on body temperature in rodents: pilot studies of the National Toxicology Program's reverberation chamber exposure system. Bioelectromagnetics. (2018) 39:190–9. 10.1002/bem.22116 29537695

[12] Falcioni L Bua L Tibaldi E Lauriola M De Angelis L Gnudi F. Report of final results regarding brain and heart tumors in Sprague-Dawley rats exposed from prenatal life until natural death to mobile phone radiofrequency field representative of a 1.8 GHz GSM base station environmental emission. Environ Res. (2018) 165:496–503. 10.1016/j.envres.2018.01.037 29530389

[13] Myung SK Ju W McDonnell DD Lee YJ Kazinets G Cheng CT. Mobile phone use and risk of tumors: a meta-analysis. J Clin Oncol. (2009) 27:5565–72. 10.1200/J.C.O.2008.21.6366 19826127

[14] Choi YJ Moskowitz JM Myung SK Lee YR Hong YC. Cellular phone use and risk of tumors: systematic review and meta-analysis. Int J Environ Res Public Health. (2020) 17:8079. 10.3390/ijerph17218079 33147845 PMC7663653

[15] EMF Academy. Lowest Radiation Cell Phones of 2022. (2022). Available online at: https://emfacademy.com/lowest-radiation-cell-phones/ (access in September 2022).

[16] Talla V Kellogg B Gollakota S Smith JR. Battery-free cell phone. Proc ACM Interact. Mobile, Wearable Ubiquitous Technol. (2017). p. 1–2. 10.1145/3090090

